# Development and function of natural TCR^+^ CD8αα^+^ intraepithelial lymphocytes

**DOI:** 10.3389/fimmu.2022.1059042

**Published:** 2022-12-07

**Authors:** Yuanyuan Gui, Hao Cheng, Jingyang Zhou, Hao Xu, Jiajia Han, Dunfang Zhang

**Affiliations:** ^1^ Department of Biotherapy, State Key Laboratory of Biotherapy and Cancer Center, West China Hospital, Sichuan University, Chengdu, China; ^2^ State Key Laboratory of Oral Diseases, National Clinical Research Center for Oral Diseases, West China Hospital of Stomatology, Sichuan University, Chengdu, China; ^3^ Precision Research Center for Refractory Diseases, Institute for Clinical Research, Shanghai General Hospital, Shanghai Jiao Tong University of Medicine, Shanghai, China

**Keywords:** intraepithelial lymphocytes (IELs), CD8αα^+^, intraepithelial lymphocytes precursors (IELps), thymus, TCRαβ^+^ CD8αα^+^ IELs, TCRγδ^+^ CD8αα^+^ IELs

## Abstract

The complexity of intestinal homeostasis results from the ability of the intestinal epithelium to absorb nutrients, harbor multiple external and internal antigens, and accommodate diverse immune cells. Intestinal intraepithelial lymphocytes (IELs) are a unique cell population embedded within the intestinal epithelial layer, contributing to the formation of the mucosal epithelial barrier and serving as a first-line defense against microbial invasion. TCRαβ^+^ CD4^-^ CD8αα^+^ CD8αβ^-^ and TCRγδ^+^ CD4^-^ CD8αα^+^ CD8αβ^-^ IELs are the two predominant subsets of natural IELs. These cells play an essential role in various intestinal diseases, such as infections and inflammatory diseases, and act as immune regulators in the gut. However, their developmental and functional patterns are extremely distinct, and the mechanisms underlying their development and migration to the intestine are not fully understood. One example is that Bcl-2 promotes the survival of thymic precursors of IELs. Mature TCRαβ^+^ CD4^-^ CD8αα^+^ CD8αβ^-^ IELs seem to be involved in immune regulation, while TCRγδ^+^ CD4^-^ CD8αα^+^ CD8αβ^-^ IELs might be involved in immune surveillance by promoting homeostasis of host microbiota, protecting and restoring the integrity of mucosal epithelium, inhibiting microbiota invasion, and limiting excessive inflammation. In this review, we elucidated and organized effectively the functions and development of these cells to guide future studies in this field. We also discussed key scientific questions that need to be addressed in this area.

## Introduction

Intestinal intraepithelial lymphocytes (IELs) are embedded within the intestinal epithelial layer of many species, including fish, pigs, mice, and humans (1, 2), although their quantity and distribution varies among species (3). These cells were initially described in 1847 as round cells within the epithelium of the small intestine and were defined as nutrition-absorbing cells (4). Later research suggested that they are predominantly composed of T cells and play a role in dealing with antigens from the intestinal lumen (4, 5). IELs were previously divided into conventional and unconventional subsets, with the former originating from CD4^+^ or T cell receptor (TCR)αβ^+^ CD8αβ^+^ T cells and migrating from peripheral lymphoid tissues, and the latter arising from CD4^-^ CD8αβ^-^ double-negative cells and migrating from the thymus (5). Further studies have identified several subsets of TCR-negative cells and revealed that IELs are a heterogeneous cell population that contains diverse TCR-positive and TCR-negative subsets (6).

TCR^-^IELs have been classified in recent years, including innate lymphoid (ILC)-like cells, iCD8α cells, and other iCD3^+^ cells (iCD8α cells are a special subtype of iCD3^+^ cells that express CD8α homodimers) (6–9). TCR^+^ IELs are classified as induced and natural IELs. Induced IELs are mostly either CD4^+^ or CD8αβ^+^, with a minority of CD8αα^+^ (6, 10); natural TCR^+^ IELs comprise TCRαβ^+^ and TCRγδ^+^ T cells along with CD8α homodimers, instead of CD4 or CD8αβ (10). TCRαβ^+^ CD4^-^ CD8αβ^-^ CD8αα^+^ (hereafter called TCRαβ^+^ CD8αα^+^ IELs) and TCRγδ^+^ CD4^-^ CD8αβ^-^ CD8αα^+^ (hereafter called TCRγδ^+^ CD8αα^+^ IELs) cells are two subtypes of natural IELs that decrease with age, also named natural CD8αα IELs, because CD8αα is regarded as their hallmark (11).

Substantial evidence indicates that CD8αα IELs share specific phenotypes, developmental pathways, migration patterns, gene profiles, and functions with other IELs subsets. Although the two CD8αα IELs subsets share multiple characteristics, and thus, can sometimes be classified into the same population, several significant differences were observed. To the best of our knowledge, TCRαβ^+^ CD8αα^+^ IELs and TCRγδ^+^ CD8αα^+^ IELs are the two major cell populations within the intestinal epithelium and account for the majority of IELs. Recent studies have also partly uncovered their role in immune surveillance, immune response, mucosal epithelial protection and restoration, immune homeostasis, systemic metabolism, and immune regulation in the local environment of the intestine. This review focuses on TCRαβ^+^ CD8αα^+^ and TCRγδ^+^ CD8αα^+^ IELs and aims to reveal the unique pathways of their development and functional characteristics.

## Classification of IELs

### TCR- IELs

**Figure 1:**
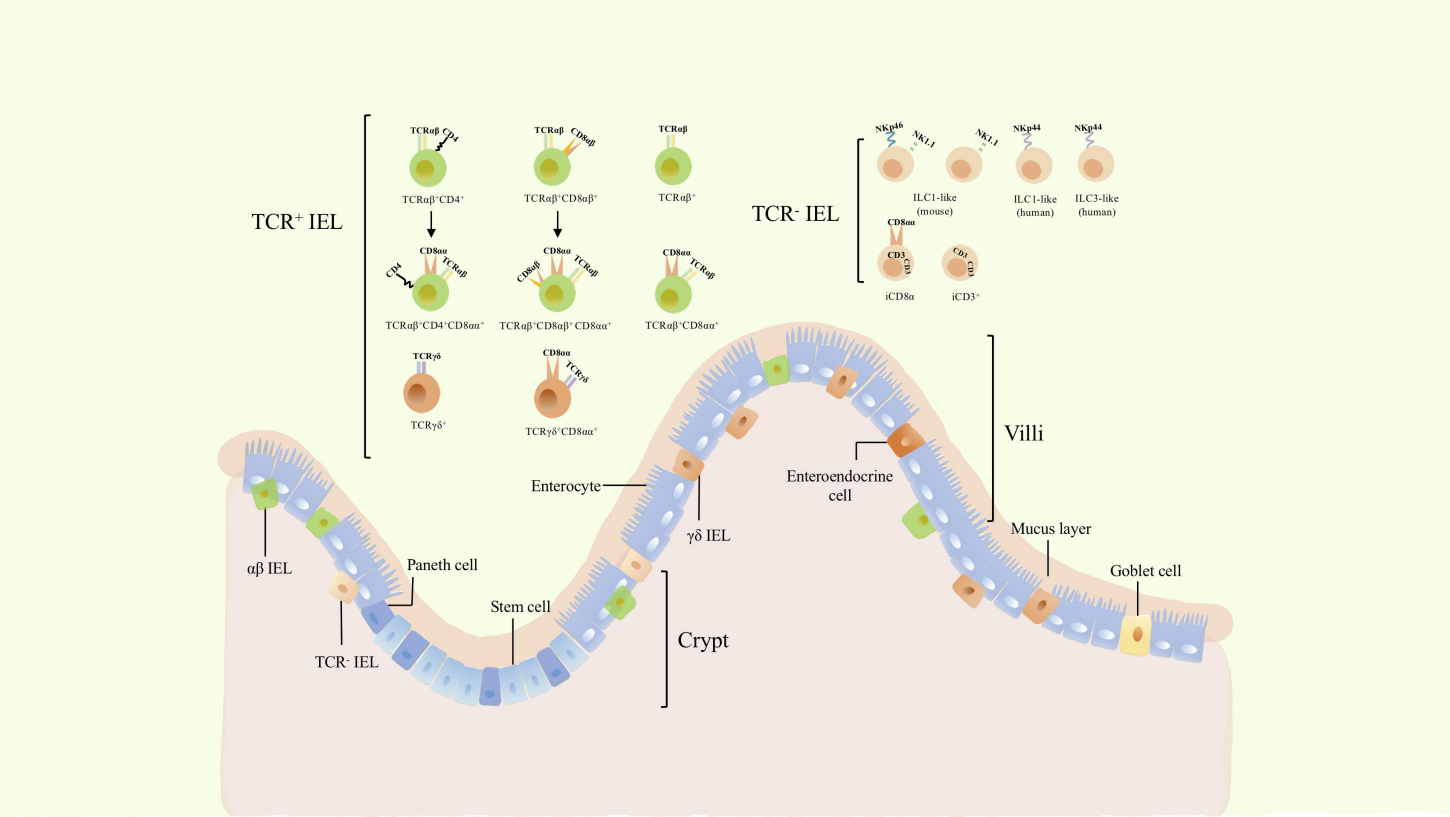
The classification and location of intraepithelial lymphocytes (IELs). Gut epithelium is composed of a single layer of enteroendocrine cells (intestinal epithelial cells). IELs are a group of heterogenous cells embedded within intestinal epithelium. Dependeing on the expression of TCR, they can be divided into TCR^+^ and TCR^-^ IELs. TCR^+^ IELs include αβ and γδ T cells. The former includes TCRαβ^+^ CD4^+^, TCRαβ^+^ CD8αβ^+^, as well as induced TCRαβ^+^ CD4^+^ CD8αα^+^ and TCRαβ^+^ CD8αβ^+^ CD8αα^+^ cells. TCRαβ^+^, TCRαβ^+^ CD8αα^+^, γδ IELs and TCR^-^ IELs cells are natural cellular subsets. γδ IELs consists of TCRγδ^+^ and TCRγδ^+^ CD8αα^+^ cells, while TCR^-^ IELs comprises ILC1-like, ILC3-like, and iCD3^+^ cells including its special subset, iCD8α.

TCR^+^ IELs have been investigated for several decades; nevertheless, TCR*^-^* IELs have been recently discovered and shown to comprise several cellular subsets (**Figure 1**). NKp44^+^ CD103^+^ ILC1 populations that express CD160 and CD101 (markers of intraepithelial lymphocyte) are embedded not only within the intestinal epithelium of humans but their counterparts have been identified in mice as cell populations expressing CD160, NKp46, and NK1.1 (8). In addition, partial CD3^-^ IELs express CD56, NKp44, IL-23R, RORγt, and gut-homing chemokine receptor CCR6, thus displaying the characteristics of three cell subsets: NK cells, ILC1, and ILC3 (12). In a subsequent study, a more comprehensive strategy for characterizing ILC was established by suggesting that these are closely associated with NK cells and are described as ILC-like cells (13).

In addition to ILC-like subsets, other special cell populations of TCR^-^ IELs have been recently identified: iCD3^+^ and iCD8α^+^ populations. iCD8α cells comprises a new innate TCR^-^ IELs population expressing CD8α as homodimers and was discovered in both humans and mice (9). Similar to TCRαβ^+^ CD8αα^+^ IELs and TCR γδ ^+^ IELs, the development of iCD8α cells also requires IL-15 and E8_I_ enhancers (9). Another subset of TCR*^-^* -IELs was further identified to reside in both humans and mice. These cells display hybrid characteristics of ILCs and T cells, express intracellular CD3, and are named iCD3 cells (7),. This evidence suggests that iCD8α cells might belong to the group of iCD3 cells (7).

### TCR^+^ IELs

TCR^+^ IELs are a well-characterized population of cells (6)and include diverse TCRαβ^+^ and TCRγδ^+^ cells (**Figure 1**). They can be classified into induced and natural IELs based on different developmental origins and phenotypes (14). Induced IELs primarily express CD4 or CD8αβ, derive from conventional TCR αβ^+^ T cells of peripheral lymphoid tissues, and include TCRαβ^+^ CD4^+^, TCRαβ^+^ CD8αβ^+^, TCRαβ^+^ CD4^+^ CD8αα^+^, and TCRαβ^+^ CD8αβ^+^ CD8αα^+^ IELs (5, 6). In contrast to induced IELs, natural IELs comprise TCRαβ^+^, TCRαβ^+^ CD8αα^+^, TCRγδ^+^, and TCRγδ^+^ CD8αα^+^ cells, and originate from TCR αβ^+^ CD4^-^ CD8αβ^-^ and TCRγδ^+^ CD4^-^ CD8αβ^-^ double-negative cells, respectively. The latter are able to migrate to the intestinal epithelium after undergoing thymic development and subsequently acquire the CD8αα phenotype (5). Furthermore, TCR^-^IELs belong to natural IELs. In addition to distinct developmental pathways, induced IELs are absent at birth and increase with age, while natural IELs are present at birth and decrease with age (5, 6). This suggests that the reduction in natural IELs may be due to an increase in induced IELs. TCRαβ^+^ CD8αα^+^ and TCRγδ^+^ CD8αα^+^ IELs are two important subsets of TCR^+^ IELs, which comprise a large proportion of IELs and play critical roles in the intestinal immune response and tolerance.

## Development of natural CD8αα^+^ IELs

### TCRαβ^+^ CD8αα^+^ IELs

**Figure 2:**
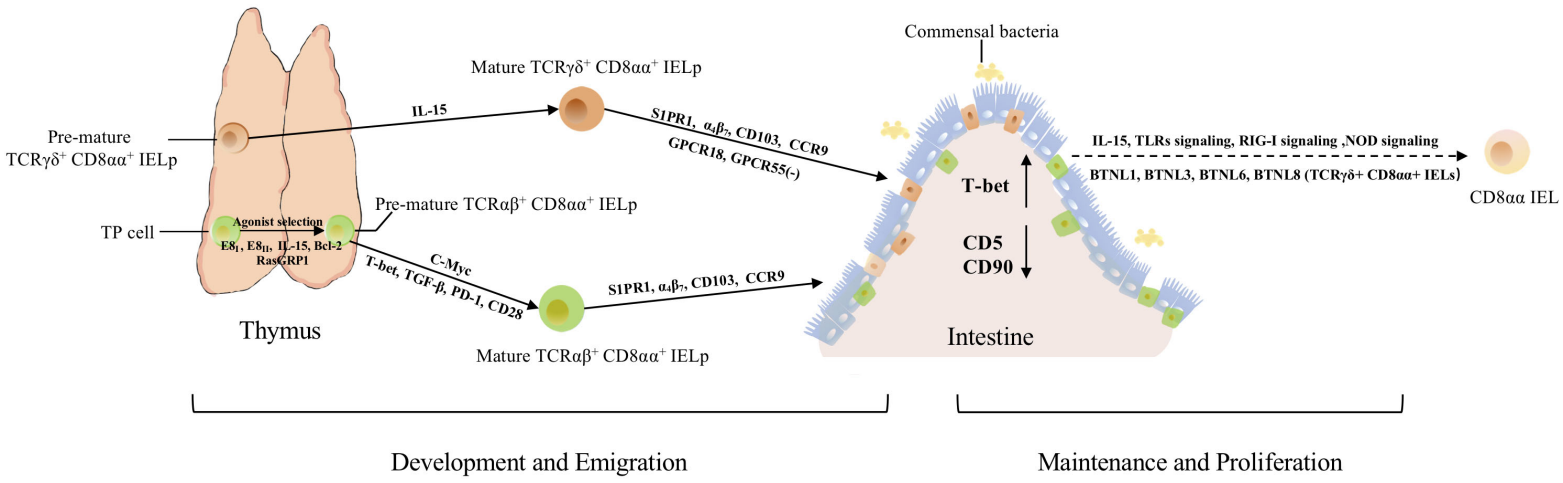
The development, migration, maintenance, and proliferation of TCRαβ^+^ CD8αα^+^ and TCRγδ^+^ CD8αα^+^ IELs. Both types arise from thymic IELps. TP cells become DN cells by regulation from E8I, E8II, IL-15, Bcl-2, and RasGRP1. E8I and E8II can suppress the expression of CD8αβ. RasGRP1 contributes to the transmission of weak TCR signals in the process of selection. Besides, c-Myc controls the development of TCRαβ^+^ CD8αα^+^ IELps *via* IL-15 and Bcl-2. After agonist selection, pre-mature TCRαβ^+^ CD8αα^+^ IELps further develop with the help of T-bet, TGF-β, and PD-1. Mature TCRαβ^+^ CD8αα^+^ IELps migrate to the intestine directly with the help of S1PR1, α4β7, CD103, and CCR9. Besides these molecules, TCRγδ^+^ CD8αα^+^ IELps also require GPCR18 and GPCR55 for localization and regulation of their accumulation. After IELps arrive in the intestine, the expression of CD5 and CD90 is downregulated, while the expression of T-bet is upregulated, exhibiting the phenotype of CD8αα. Meanwhile, the crosstalk between commensal bacteria, IECs, and CD8αα IELs contributes to the maintenance and proliferation of CD8αα cells, *via* NOD2 signaling, TLRs signaling, RIG-I signaling, IL-15, and other signaling pathways. In addition, BTNL1, BTLN3, BTNL6 and BTNL8 could promote the maturation and expansion of γδ IELs.

TCRαβ^+^ CD8αα^+^ IELs are first identified in mice and the existence of them in humans remains controversial (4). Some studies suggested that this population is present in gestation and rare in adult humans (4, 6). This group of cells are one of the predominant populations in diverse IELs subsets. Nonetheless, TCRαβ^+^ CD8αα^+^ IELs have a contentious origin. It was initially thought that development and differentiation occur in the thymus, but further studies reported the presence of TCRαβ^+^ CD8αα^+^ IELs in irradiated, neonatally thymectomized, and athymic mice, thus suggesting that not all IEL populations are developed by a functional thymus (15). In subsequent studies, some researchers proposed that TCRαβ+ CD8αα+ IELs are generated independently of the thymus, whereas the generation of other subsets of IELs, including CD8 αβ^+^ and CD4^+^CD8αα^+^, is thymus-dependent (16). Meanwhile, precursors of CD8αα^+^ IELs are present in the gut, making some researchers believe that the development and differentiation of CD8αα^+^ IELs occur in the intestinal region (17). In subsequent studies on the identification of iCD8α IELs, the hypothesis that the precursors of conventional IELs were TCR^-^ CD8α^+^ cells in the intestinal epithelia, was controversial. Furthermore, substantial evidence has indicated that both TCRαβ^+^ CD8αα^+^ and TCRγδ^+^ CD8αα^+^ IELs originate from thymic cells, suggesting that the potential precursors reside in double-negative thymocytes. Meanwhile, athymic mice had a lower number of TCRαβ^+^ CD8αα^+^ IELs which could be restored after transplanting the fetal thymus, confirming that the majority of TCRαβ^+^ CD8αα^+^ IELs arose from the thymus, while the extrathymic pathway may also provide such cells in adults (**Figure 2**) (18–20).

Until now, thymus-dependent development of TCRαβ^+^ CD8αα^+^ IELs was mostly agreed upon, as the thymus is an important organ for self-antigen recognition and selection of T cells. After induction by TCRβ, pre-TCR-CD3 signaling, and other signaling molecules, a small fraction of CD4^+^ CD8αβ^+^ CD8αα^+^ thymocytes (i.e., TP cells), were the post-selection precursors of TCRαβ^+^ CD8αα^+^ IELs (21), which retained the expression of CD8αα at the stage of positive selection (21). The noncoding region of *Cd8* gene, E8_I_, as well as the combination of E8_I_ and E8_II_ (both CD8α enhancers) are also involved in the expression of CD8αα and the suppression of the expression of CD8αβ in immature thymocytes (22–24). Recently, the specific precursors of TCRαβ^+^ CD8αα^+^ IELs have been identified. Two subsets of precursors of TCRαβ^+^ CD8αα^+^ IELs (hereafter called IELps) were identified from the TCRβ^+^ CD5^+^ CD122^+^ H-2Kb^+^ CD4^-^ CD8^-^ thymocytes: PD-1^+^ T-bet^-^ cells (hereafter called PD-1^+^ IELps) and T-bet^+^ PD-1^-^ cells (hereafter called T-bet^+^ IELps) (25). PD-1^+^ IELps are localized in the cortex and restricted by classical major histocompatibility complex (MHC) molecules. They are nascent and self-reactive, whereas T-bet^+^ IELps are located in the medulla and restricted by non-classical MHC I molecules, and their number increases with age (25). Meanwhile, only T-bet^+^ IELps expressed the memory marker CD44 and chemokine receptor CXCR3, while neither PD-1^+^ IELps nor T-bet^+^ IELps expressed CCR7 (25). Although two kinds of IELps could give rise to TCRαβ^+^ CD8αα^+^ IELs, evidence indicates that T-bet^+^ IELps are preferentially retained in the thymus, and PD-1^+^ IELps are the main precursors of TCRαβ^+^ CD8αα^+^ IELs (25). In a subsequent study, CD122^+^ PD-1^+^ α4β7^+^ CD103^-^ IELps and CD122^+^ PD-1^-^ $\alpha_{4}\beta_{7}^{-}$ CD103+ IELps were identified, and it was proposed that the former subset was congruent with PD-1^+^ IELps, whereas the latter was represented by T-bet^+^ IELps (26). This further proves the presence of two types of thymic IELps. In a recent study, researchers found a group of killer innate-like T cells (ILTCks) could mediate cancer immunity, whereas showed αβILTCk-TCR expressing thymocytes co-expressed PD-1 and CD122, which is similar to IELps, revealed the αβILTCk-TCR thymocytes could also differentiate into IELs (27).

Furthermore, IL-15 might participate in the differentiation of TP precursors (21). The maturation of IELps is accompanied by the upregulation of MHC class I molecules H-2Kb and CD122 (25, 28). Jiang et al. proposed that c-Myc regulates the development of IELps *via* IL-15- and Bcl-2-dependent survival (29). Agonist selection and IL-15 receptor signaling can induce T-bet expression, indicating that T-bet, TGF-β, and PD-1 are all involved in the development of CD8αα^+^ IELs (**Figure 2**) (25, 30, 31). The development of thymic IELps does not depend on IL-15 (25, 32). Although researchers have defined several characteristics of IELps, their maturation, localization, and emigration patterns are still not fully understood.

The development of different T cell lineages requires TCR signals. Similar to regulatory T cells, TCRαβ^+^ CD8αα^+^ IELs are self-reactive and require exposure to self-agonists in the thymus (26, 33). PD-1^+^ IELps express PD-1, CD69, Nur77, and Egr2, display signs of elevated TCR signaling (34), and are capable of self-reactivity after undergoing positive agonist selection (35, 36). However, the high affinity of TCRs for self-antigens or MHC is removed to maintain self-tolerance. The number of PD-1^+^ IELps increased in Bim-deficient mice, suggesting that IELps may also be produced by clonal deletion (37). However, the mechanism by which IELps escape deletions is not fully understood. Some DP thymocytes survive by downregulating the expression of CD8β and upregulating the expression of CD8αα, CD8αα^+^ cells, which would also activate an altered gene expression program (21, 38–41). These results indicate a possible mechanism by which IELps survive. Furthermore, RAS Guanyl Releasing Protein 1 (RasGRP1), a Ras activator required to transmit weak TCR signals, is also an essential molecule for the survival of TCRαβ^+^ CD8αα^+^ IELps during agonist selection (26). In addition, CD28-deficient mice have more PD-1^+^ IELps (25), and PD-1 can inactivate CD28 signaling (42), suggesting that PD-1 and CD28 may play roles in the survival and differentiation of IELps. Meanwhile, the anti-apoptotic protein Bcl-2 promotes the survival of IELps and TCRαβ^+^ CD8αα^+^ IELs by antagonizing Bim (43).

Although recent evidence has shed light on the development of TCRαβ^+^ CD8αα^+^ IELs, the different signals, gene programs, and molecules involved in the development of these cells are not fully understood.

### TCRγδ^+^ CD8αα^+^ IELs

γδ T cells reside in various organs such as the intestine, skin, vagina, gingiva, uterus, and tongue (44–48). Meanwhile, more γδ T cells reside in the intestinal intraepithelial tissue than in other tissues. TCRγδ^+^ CD8αα^+^ IELs are present in both humans and mice. In humans, only 13% of IELs are γδ T cells (49), whereas in mice, the proportion of γδ T cells is around 50-60% (6, 10, 49, 50). Most γδ IELs expressed CD8αα homodimers (hereafter TCRγδ^+^ IELs referred to both TCRγδ^+^ IELs and TCRγδ^+^ CD8αα^+^ IELs).

The TCR specificity of TCRγδ^+^ CD8αα^+^ IELs is unknown, but seems similar to that of conventional peripheral γδ T cells (6). Comparable to TCRαβ^+^ CD8αα^+^ IELs, the origin and development of TCRγδ^+^ CD8αα^+^ IELs have been controversial (**Figure 2**). Previous studies indicated that they developed in the absence of the thymus, while others proposed they originate from the thymus. Although the thymic precursors and development of TCRγδ^+^ CD8αα^+^ IELs remain poorly understood, their development and differentiation are very similar to those of TCRαβ^+^ CD8αα^+^ IELs, for example, in terms of the expression of CD8αα as well as the suppression of CD8β. Additionally, they may require the same molecules and programs to develop, differentiate, and survive. Nonetheless, in contrast to TCRαβ^+^ CD8αα^+^ IELs, the repertoire and development of TCRγδ^+^ CD8αα^+^ IELs seemed to be unaffected by MHC antigens and RasGRP1 (26), and were independent of microbial and food antigens (51).

Butyrophilin-like proteins (BTNL; members of the B7 superfamily of costimulatory receptors) are expected to act as co-stimulators of IEL receptors. However, the functions of BTNL members have not yet been elucidated. BTNL1, BTNL3, BTNL6, BTNL8, BTN3A1, BTN3A2, and Skint1 are involved in the regulation of TCR γδ ^+^ cells, with BTNL1, BTNL4, and BTNL6 being widely expressed in the mouse gut (52). The number of TCRγδ^+^ IELs is reduced in Btnl1^-/-^ mice, suggesting that BTNL1 expressed by the epithelial cells of small intestinal villi, promotes the maturation and expansion of TCRγδ^+^ IELs (51). In addition, BTNL1 together with BTNL6 can induce TCR-dependent stimulation of γδ^+^ T cells (51). Further experiments confirmed that BTNL6 and BTNL1 are required for the development of TCR γδ ^+^ IELs (53). Additionally, BTNL3 and BTNL8 expressed in the human gut epithelium can regulate the development of TCR Vγ4 (51). Furthermore, Skint, a Btnl gene expressed by thymic epithelial cells and suprabasal keratinocytes, drives the maturation of progenitors of dendritic epidermal T cells (DETCs) (54, 55), suggesting that this gene may also facilitate the maturation of TCRγδ^+^ IELs. However, this is debatable, because Skint genes are only expressed in γδ T cells residing in the skin and thymus (55). Collectively, these results suggest that intestinal epithelial cells (IECs) may facilitate the development and function of TCRγδ^+^ CD8αα^+^ IELs.

## Migration and maintenance of natural CD8αα^+^ IELs

Conventional T cells arise from lymphoid precursors, which are derived from pluripotent stem cells in the marrow and migrate to the thymus. In the thymus, within the cortex, T cell progenitors undergo positive selection and migrate to the medulla for further differentiation, selection, and maturation, which imply a delicate regulatory program. For example, the expression of CCR7 is upregulated to facilitate migration. In addition, TGF-β-activated kinase 1 (TAK1) facilitates the functional maturation of T cells, and NF-κB signaling is required for cell proliferation and egress (56, 57). After acquiring the competence to proliferate and migrate, T cells move from the perivascular spaces into the vasculature in response to sphingosine-1 phosphate binding to sphingosine-1 phosphate receptor 1 (S1PR1; G-protein-coupled receptor) (58–63). Like conventional T cells, IELps also express S1PR1, indicating that they may employ a similar mechanism of egress from the thymus (**Figure 2**). Mature IELps express S1PR1 (59, 62, 63), confirming the hypothesis that IELps depend on S1PR1 to enable thymic egress (64). After migrating from thymus to vasculature, lymphocytes roll alone the endothelial cells, then adhere to them and migrate across the endothelium to emigrate from the vasculature into tissues (65). Previous studies exhibited that α_4_β_7_ is a receptor to MAdCAM-1, while MAdCAM-1 is expressed by mucosal venules to help lymphocyte traffic into Peyer’s patches and the intestinal lamina propria (LP), suggested α_4_β_7_ mediates the adherence of IELs to intestinal epithelial (65–67). Integrins α_4_β_7_ and α_E_β_7_ (i.e., CD103, a hallmark of tissue-resident T cells), CD122, CD160, and 2B4 are common molecules associated with gut-homing and retention of cells (48, 66, 68–71); the expressions of α_4_β_7_, CD103, and CCR9 direct competent IELps migrate, entry and firmly attach to the gut epithelium (**Figure 2**) (14, 25, 30, 72, 73). Meanwhile, recent study showed that transcription factor LRF could promote the expression of integrin α4β7, control the late differentiation and facilitate the gut-homing process of CD8αα IELp (74). Meanwhile, mice lacking the vitamin D receptor showed low expression of CCR9 (75), indicating that vitamin D is also a factor affecting the migration of CD8αα^+^ IELs. Furthermore, orphan receptor G protein-coupled receptor 18 (GPCR18) is required for the localization of CD8αα IELs, especially TCRγδ^+^ CD8αα^+^ IELs (**Figure 2**) (76). GPCR 55 negatively regulates the accumulation of TCRγδ^+^ CD8αα^+^ cells (**Figure 2**) (77).

During the agonist-selection process, TP cells express high levels of CD5 and CD90, indicating that these cells receive high TCR activation signals and then become DN αβT cells (30). After CD8αα^+^ IELs arrive in the gut, the expression of CD5 and CD90 is downregulated and the expression of CD103 and CD8αα is upregulated, and CD8αα^+^ IELs become resident cells (**Figure 2**) (21, 30, 78). Meanwhile, CD8αα^+^ IELs also upregulate the expression of T-bet, which could induce the expression of CD8αα homodimers (**Figure 2**) (30). IL-15 is a critical molecule that mediates the expression of T-bet and CD5, and there is evidence that IL-15 is involved in the maintenance and expansion of CD8αα^+^ IELs instead of their induction (**Figure 2**) (21).

The development, survival, and maintenance of CD8αα^+^ IELs is affected by diverse molecules and factors (**Figure 2**). Exposure to external food antigens or pathogens and different gut environments can shape and maintain CD8αα^+^ IELs. Gut bacteria can shape the differentiation of diverse T cells (79–84). Cervantes-Barragan et al. showed that *Lactobacillus reuteri* (L. reuteri) produced indole derivatives of tryptophan which activate the aryl hydrocarbon receptor, allowing downregulation of the expression of T-helper-inducing POZ/Krueppel-like factor (ThPOK), which is implicated in the differentiation of CD4^+^ CD8αα^+^ double-positive IELs (DP IELs) (85). This result suggests that ThPOK plays a role in regulating the expression of CD8α and that microbial factors or specific diets could promote the differentiation and maintenance of IELs.

NOD2 signaling helps maintaining the homeostasis of CD8αα+ IELs *via* the recognition of gut microbiota and IL-15 production (86). This further demonstrates that the gut microbiota promotes the retention of CD8αα^+^ IELs. Meanwhile, Yu et al. suggested that MyD88-dependent signaling contributed to the maintenance of the number of TCRαβ^+^ CD8αα^+^ IELs and TCRγδ^+^ IELs *via* IL-15 production, which was influenced by the interaction between commensal bacteria and IECs *via* TLRs signaling (87). As c-Myc regulates the development of IELps *via* IL-15, and IL-15 mediates the expression of T-bet to induce the expression of CD8αα homodimers and help maintain the homeostasis through NOD2 and MyD88-dependent signaling, IL-15 is considered to be involved in the development and maintenance of TCRαβ^+^ CD8αα^+^ IELs. Meanwhile, as another study showed that IECs, macrophages and DCs in the intestine could express IL-15 (86), and enterocytes express BTNLl1, BTNL3, BTNL6, and BTNL8 of the BTNL family to promote the expansion of TCRγδ^+^ CD8αα^+^ IELs (51), these results indicated that IECs and other cells in intestine may help the maintenance and expansion of TCRαβ^+^ CD8αα^+^ and TCRγδ^+^ CD8αα^+^ populations *via* expression of IL-15 and of BTNL molecules. Commensal viruses and retinoic acid-inducible gene I (RIG-I) signaling are essential for the homeostasis of IELs (88). Furthermore, the thymus leukemia antigen, which is confined to the surface of IECs, functions as an effective effector in modulating the IEL response (89). These results suggested that multiple cells and viruses in the intestine contribute to the survival and maintenance of CD8αα^+^ IELs.

Konijnenburg et al. revealed that the dynamic localization and distribution, migration, scanning patterns, and energy utilization of TCRγδ^+^ IELs are driven by microbial density through the sensing of IECs (3), which is a consequence of epithelial-immune crosstalk. In a subsequent study, Jia et al. identified commensal bacteria that contributed to γδ IELs surveillance (90). Furthermore, the development and homeostasis of TCRαβ^+^ CD8αα^+^ IELs requires β2m expression, not of classical class I molecules K and D (70). Moreover, a recent study indicated that the development and maintenance of CD8αα^+^ IELs partly depend on low oxygenic conditions (91).

## Function of various IELs in gut epithelium

**Figure 3:**
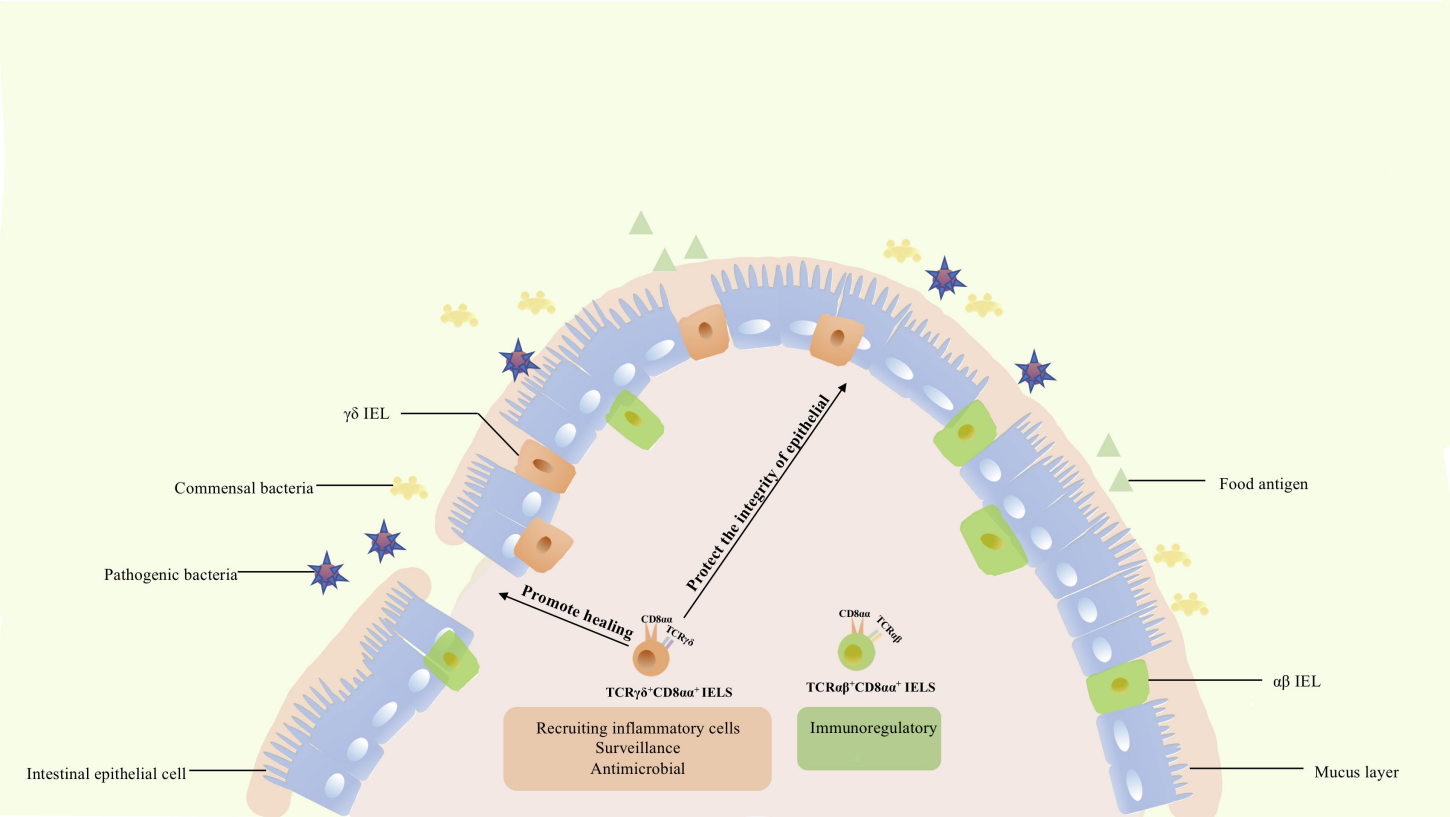
The functions of CD8αα IELs. TCRαβ^+^ CD8αα^+^ IELs express Ly49A, Ly49C, Ly49E, and other genes of the Ly49 family, as well as 2B4, fibrinogen-like protein-2, TGF-β3, and LAG-3, being involved in immune regulation. In contrast to TCRαβ^+^ CD8αα^+^ IELs, TCRγδ^+^ IELs express TGFβ1, TGFβ3, prothymosin β4, heat shock proteins, chemokine KC, and βig-h3, being involved in injury healing and protection of the integrity of epithelium. Meanwhile, these cells could also express cytokines KC, IL-1β, MIP2α, Cxc19, and Cxc116 thus recruiting various inflammatory cells. Besides, they are characterized by a specific dynamic pattern to surveil and respond to pathogen invasion, undertaking diverse roles in the intestine.

The gut epithelium is a unique immunological compartment that is in contact with numerous external microorganisms and environmental antigens and as well as with the internal environment. The gut epithelium comprises a single layer of IECs, with diverse IELs embedded between these cells, and provides the first line of defense. This suggests that these cells may undertake potentially essential functions, despite the small total proportion of IELs. Considering this characteristic, the gut mucosal immune system requires a delicate program to respond to pathogens, while maintaining tolerance to innocuous antigens. In mice, studies showed that IELs increase in the late disease process of enteropathies such as CeD, graft vs. host disease, allograft rejection, autoimmune (4). In human, TCRαβ^+^ CD8αβ^+^ IELs and innate-like IEL lacking surface TCR expression were involved in the development of villous atrophy in patients with refractory CeD (4). CD8αα homodimers decreased antigen sensitivity of the TCR and acted as repressors to negatively regulate T cell activation (92). CD8αα IELs are related to inflammatory bowel disease (IBD) and infection and play a critical role in protection against pathogens, as well as in controlling bacterial overgrowth. This indicates their involvement in the promotion of mucosal defense and epithelial homeostasis (89, 93–96). Besides, recent study showed that integrin β7 deficiency protects mice from metabolic syndrome and against atherosclerosis, whereas IELs in the small intestine had the highest expression of β7, revealed that β7^+^ natural IELs could modulate systemic metabolism and accelerate the progression of cardiovascular disease (97). Although most of these functions are shared, the functions of the different subsets of IELs differ slightly (**Figure 3**).

### Functions of TCRαβ^+^ IEL

The function of TCRαβ^+^ CD8αα^+^ IELs has not been completely elucidated. In general, IELs expressing TCRαβ can respond to pathogens. Global analysis revealed that this population expressed NK receptor-related genes, such as Ly49A, Ly49C, and Ly49E of the Ly49 family, and genes that were expected to down-modulate their reactivity (70). These cells also express fibrinogen-like protein-2, TGF-β3, LAG-3, and genes associated with corresponding inhibitory or activation functions, such as 2B4 (70). TCRαβ^+^ CD8αα^+^ IELs and NK cells share similar characteristics, and TCRαβ^+^ CD8αα^+^, TCRαβ^+^ CD8αβ^+^, and TCRγδ^+^ CD8αα^+^ IELs have significantly different functions. TCRαβ^+^ CD8αα^+^ IELs might have suppressive and regulatory roles. Besides, this cellular population prevents induced colitis, a role mediated by IL-10. This method of protection is unique and differs from that of TCRγδ^+^ and TCRαβ^+^ CD8αβ^+^ IELs (70, 98). Collectively, these results indicate that TCRαβ^+^ CD8αα^+^ IELs contributes to the maintenance of intestinal immunity and immune regulation.

### Functions of TCRγδ^+^ IEL

TCRγδ^+^ IELs were scattered predominantly in the central and upper locations of the villi (3). Although TCRγδ^+^ and TCRαβ^+^ CD8αα^+^ IELs share similar developmental pathways and expression of specific genes, these subsets are significantly different. In contrast to TCRαβ^+^ CD8αα^+^ IELs, the TCRγδ^+^ population did not show a significantly high expression of NK receptor-related genes or of the other genes mentioned previously (70).

Unlike αβ T cells, γδ T cells commonly contribute to the maintenance and restoration of body-surface integrity. Boismenu et al. proposed that activated TCRγδ^+^ IELs produce keratinocyte growth factor (an epithelial cell growth factor belonging to the fibroblast growth factor family) and stimulate the differentiation, regeneration, and migration of epithelial cells, whereas TCRαβ^+^ IELs do not (99). Furthermore, a substantial amount of TCRγδ^+^ IELs was enriched around the injured region in dextran sodium sulfate (DSS)-induced mouse colitis (100). TCRγδ^+^ IELs upregulated the expression of cytoprotective factors such as heat shock proteins, chemokine KC, and βig-h3 to promote keratinocyte proliferation and wound healing during DSS treatment (101). In addition, TCRγδ^+^ IELs secrete TGFβ1, TGFβ3, and prothymosin β4 which protect the intestinal epithelium (14). These studies further confirmed that TCR γδ^+^ IELs resolved inflammatory lesions by secreting multiple factors. However, although studies have shown that TCRγδ^+^ IELs help maintain and restore the integrity of intestinal epithelia in IBD (100, 102), the function of TCRγδ^+^ IELs in this pathology is not fully understood. TCRγδ^+^ IELs also secrete proinflammatory factors which can induce or aggravate colitis (103, 104). Park et al. showed that activation of TCRγδ^+^ IELs by commensal bacteria induces spontaneous colitis (105). Nevertheless, this also indicates that T regulatory cells could suppress TCRγδ^+^ IELs *via* IL-10 to maintain intestinal homeostasis (105).

In addition, TCRγδ^+^ IELs upregulated the expression of chemotactic molecules such as cytokines KC, IL-1β, MIP2α, and Cxc19, for various inflammatory cells, and the expression of microbial pattern recognition receptors such as TLR1 and CD4 in DSS-induced colitis (101). Meanwhile, they are accompanied by increased complement components 1qa, 1qb, and lysozyme, which are bactericidal proteins, and by increased expression of RegIIIγ (a pancreatitis-associated protein) (101). MyD88 is also required for regulation of RegIIIγ expression, and commensal bacteria could regulate the response of TCRγδ^+^ IELs to mucosal damage through MyD88-dependent and MyD88-independent pathways (101, 106). TCRγδ^+^ IELs could also recruit inflammatory cells, respond to bacteria, and be associated with commensal bacteria. Activated TCRγδ^+^ IELs could limit bacterial penetration of resident microbiota or new organisms from the environment (106).

In addition, several studies have revealed the cytotoxic properties of activated TCRγδ^+^ IELs. These cells produce interferons, TNF-α, and antimicrobial proteins in response to viral or bacterial infections (1, 107). At the same time, the immune surveillance of TCRγδ^+^ IELs follows a dynamic migration pattern: they survey pathogen invasion by shifting along the basement membrane, migrate into the lateral intercellular space between two adjacent enterocytes and change the pattern when pathogen invasion occurs (48). Additionally, these cells facilitate tumor necrosis factor-mediated shedding of apoptotic enterocytes with the help of CD103-mediated extracellular granzyme release (108).

Collectively, although the functions and detailed molecular mechanisms of TCRγδ^+^ IELs have not been fully defined, current evidence indicates their roles in preserving and restoring the integrity of the intestinal epithelium, recruiting inflammatory cells, surveilling, responding to enteric infection, maintaining mucosal homeostasis, and facilitating pathological epithelial cell shedding. These functions indicate the importance and delicate regulatory traits of TCR γδ ^+^ IELs.

## Conclusion and unanswered questions

The gut is an essential nutrient absorption organ that directly encounters multiple antigens in the gastrointestinal tract and contains various immune cells with distinct functions and distributions. IELs are a small number of heterogeneous cells residing in the intestinal epithelium, undertaking the role of the first line of defense of the immune system. Their functions also include maintaining immune homeostasis, other possible competencies. Besides, studies exhibited IELs are associated with multiple disease such as CeD, tropical sprue and parasite infections. Natural TCRαβ^+^ CD8αα^+^ and TCRγδ^+^ CD8αα^+^ IELs are two special populations of IELs that exhibit phenotypes and characteristics that are different from conventional T cells or other subsets of IELs. TCRαβ^+^ CD8αα^+^ IELs are capable of immune regulation, whereas TCRγδ^+^ CD8αα^+^ IELs can protect the integrity of intestinal epithelia, heal injured mucosal epithelia, maintain homeostasis of the resident microbiota, inhibit microbiota invasion, respond to pathogens, and limit excessive inflammation. Meanwhile, recent study revealed the role of natural IELs in dietary metabolism, showed the potential research value of these cells. In brief, a number of studies have highlighted the importance of TCRαβ^+^ CD8αα^+^ and TCRγδ^+^ CD8αα^+^ IELs, indicating the possibility of taking advantage of these cells to strengthen the understanding of intestinal immunity, metabolism and cure diverse associated illnesses or infections.

However, the development, function, gene profiles of these cells, as well as the regulatory mechanisms underlying their effect against different conditions require further exploration. For instance, although previous studies of TCRαβ^+^ CD8αα^+^ IELs identified two thymic progenitors and revealed their distinct features, migrating patterns, and some specific gene profiles, the proportions and potential functional or phenotypic differences between the two IELps are not fully understood. TCRαβ^+^ CD8αα^+^ and TCRγδ^+^ CD8αα^+^ IELs have various roles under normal or infectious/inflammatory conditions, their existence being essential in organisms. However, the specific molecules regulating their function are not clear, although several critical transcription factors, cytokines, chemokines, and other molecules involved in their development, maturation, migration, and function, were identified. These unanswered questions should be the focus of future research.

## Author contributions

YG drafted the manuscript. HC, JZ, and HX edited the manuscript. JH and DZ supervised the work and edited the manuscript. All authors contributed to the article and approved it for publication.
